# Detection-Driven Gaussian Mixture Probability Hypothesis Density Multi-Target Tracker for Airborne Infrared Platforms

**DOI:** 10.3390/s25113491

**Published:** 2025-05-31

**Authors:** Mingyu Hong, Jiarong Wang, Ming Zhu, Shenyi Cao, Haitao Nie, Xiangdong Xu

**Affiliations:** 1 Changchun Institute of Optics, Fine Mechanics and Physics, Chinese Academy of Sciences, Changchun 130033, China; hongmingyu20@mails.ucas.ac.cn (M.H.); zhuming@ciomp.ac.cn (M.Z.); niehaitao@ciomp.ac.cn (H.N.); xuxiangdong21@mails.ucas.ac.cn (X.X.); 2 University of Chinese Academy of Sciences, Beijing 100049, China; 3 Faculty of Electronic and Information Engineering, Xi’an Jiaotong University, Xi’an 710049, China; caoshenyi@stu.xjtu.edu.cn

**Keywords:** infrared object, UAV, target tracking, object detection, yolov10, GM-PHD filter

## Abstract

Recent advancements in the unmanned aerial vehicle remote sensing field have highlighted the effectiveness of infrared sensors in detecting and tracking time-sensitive ground targets, particularly within the domain of early warning and surveillance. However, the limitations inherent in airborne infrared platforms can lead to irregular imaging and inadequate textural features. This study presents a multi-object tracking system specifically designed for weak-textured infrared targets, aimed at enhancing detection accuracy and tracking stability. Initially, improvements are made to the YOLOv10 model through the incorporation of modules such as DSA, c2f_fasterblock, and NMSFree, which collectively enhance detection accuracy and robustness for weak-textured targets. Subsequently, the detection results are employed in conjunction with GM-PHD tracking, enabling rapid and stable target tracking. The proposed methodology demonstrates a 2.3% improvement in detection accuracy and a 3.8% increase in recall when assessed using publicly available infrared tracking datasets. Notably, the key tracking metric, MOTA, achieves a value of 90.7%, while the IDF1 score reaches 94.6%. The findings from the experiments indicate that the proposed algorithm surpasses current methodologies regarding effectiveness, accuracy, and robustness in the context of infrared multi-target tracking tasks, thereby meeting the requirements associated with airborne infrared target tracking tasks.

## 1. Introduction

**Figure 1:**
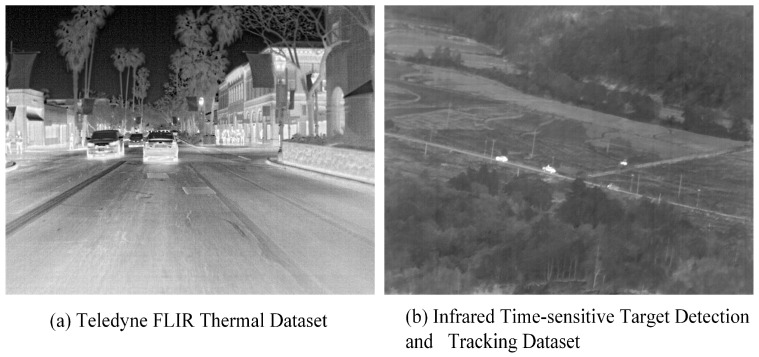
The visual differences between weak-texture infrared targets and traditional infrared targets. The comparison shows that tracking infrared weak-texture targets faces challenges such as high detection difficulty and difficulty in distinguishing occlusions.

The technology of infrared target tracking has emerged as a significant area of research within fields such as space exploration and military reconnaissance [1]. This technology is applicable in nighttime and adverse weather conditions and can be utilized for various purposes, including border security, pedestrian and obstacle detection, fire warning, life search and rescue operations, body temperature monitoring, drone inspections, and equipment fault detection. With advancements in infrared sensor technology, its role in intelligence and automation is expected to expand, thereby enhancing safety and efficiency while simultaneously fostering further development in the civilian market. A notable advantage of infrared detection lies in its resilience to adverse weather conditions and variations in lighting, which allows for continuous operation. Since it passively captures infrared radiation emitted by objects, it does not actively transmit signals, rendering it undetectable—an advantageous characteristic for target tracking [2]. However, airborne infrared platforms face considerable limitations due to restricted computational resources and the requirement to capture target images from considerable distances, where the targets may occupy only a minimal number of pixels [3,4], as depicted in Figure 1. This situation is further complicated in comparison to conventional infrared targets by interference from cloud cover and thermal noise emanating from the ground environment. As a result, these factors lead to challenges such as diminished resolution, loss of texture, and reduced signal-to-noise ratios, thereby complicating the processes of infrared target detection and tracking.

To address the challenges associated with infrared target detection, researchers have developed a variety of algorithms over the past several decades. Early methodologies, which included spatial filtering and contrast detection algorithms [5,6,7,8,9,10], primarily focused on the characteristics of target pixels. However, these approaches faced significant precision issues in complex environments. The advent of deep learning has significantly improved target detection performance, thereby facilitating the advancement of Detection-Based Tracking (DBT) methods [11,12,13,14,15,16,17]. DBT entails the detection of objects in each frame and their subsequent association based on estimated instance similarity. Effective target detection algorithms provide robust spatial proximity cues across consecutive frames, which are assessed using metrics such as Intersection-over-Union (IoU) or center distance. Despite these advancements, current DBT methods still encounter difficulties with weak-texture targets and occlusions. The pursuit of enhanced precision has resulted in the development of more sophisticated and comprehensive networks for target recognition and tracking. However, this increased accuracy often comes at the expense of efficiency, posing challenges for the implementation of complex network models in industrial applications such as automation, object counting, and video surveillance, primarily due to cost and hardware constraints.

With the widespread application of ChatGPT-3.5, its core architecture, the Transformer, has gradually been integrated into object tracking algorithms such as TransTrack [18], TrackFormer [19], and SwinTrack [20]. Utilizing the self-attention mechanism, the Transformer effectively models global contextual relationships, demonstrating strong robustness and precise tracking performance, particularly in scenarios involving long-term dependencies and complex interactions. However, these methods exhibit high computational complexity, which limits their real-time performance. Additionally, they typically require large-scale datasets for training, imposing stringent hardware deployment requirements, especially the need for high-end GPU support. When deployed on edge devices, these algorithms encounter bottlenecks in inference speed and excessive memory usage. While detection speed can be enhanced by utilizing lightweight Transformers [21], this improvement may come at the expense of tracking accuracy and robustness. Furthermore, current common edge devices still offer limited optimization support for Transformer algorithms. In contrast, tracking algorithms based on YOLOv10n [22] provide advantages in inference speed and hardware deployment, making them particularly suitable for efficient object tracking in resource-constrained environments.

In response to the challenges associated with tracking infrared weak-texture targets, this study proposes a multi-object tracking system that consists of two primary stages: single-frame target detection and tracking via the Gaussian Mixture Probability Hypothesis Density (GM-PHD) filter [23]. The single-frame target detection stage is based on the YOLOv10n model, which is specifically trained without non-maximum suppression (NMS) to mitigate inference delays that typically arise during end-to-end deployment. To overcome the limitations posed by infrared targets that exhibit minimal distinct shape and texture features, a deep–shallow attention (DSA) module is introduced. This module facilitates the network’s focus on salient features while effectively suppressing redundant and irrelevant background information, thereby enhancing the accuracy of target detection. Following the detection phase, the GM-PHD filter is utilized for multi-object tracking, adeptly managing complex scenarios characterized by uncertain observation data and fluctuating target numbers. Furthermore, an image structural similarity (SSIM) module [24] is integrated to extract structural information from the target’s surrounding environment, which contributes to a reduction in computational complexity and an increase in target association speed. By integrating the weak-texture infrared target detection model (WTI-YOLO), which is based on the YOLOv10n framework, with the GM-PHD tracking algorithm, this study presents an effective solution for the multi-object tracking of infrared weak-texture targets, fulfilling the criteria for accuracy while ensuring real-time performance. This integration provides robust technical support for practical applications in related fields. The proposed tracking system was validated using a publicly available infrared time-sensitive target detection and tracking dataset [25], with results demonstrating that the algorithm is capable of effectively detecting and tracking targets in real-time.

The primary contributions of this study can be articulated as follows:

1. The development of a multi-object tracker for infrared weak-texture targets integrates a YOLOv10-based target detection model with the GM-PHD tracking algorithm. This integration provides high inference speed and detection accuracy, making it suitable for real-time tracking tasks on airborne infrared platforms.
2. The integration of a novel deep and shallow attention module within the neck network of the WTI-YOLO model employs the RFA module to prioritize detailed features from the shallow network, while simultaneously extracting high-level semantic information from the deep network through the EMCA module. This approach significantly improves the detection accuracy for targets with weak textures.
3. The incorporation of the C2f_FasterBlock module serves to optimize WTI-YOLO, resulting in a substantial reduction in model parameters and computational complexity. This enhancement facilitates the model’s operation at speeds that align with the practical task requirements of airborne devices.
4. The selection of the GM-PHD filter for multi-object tracking is informed by recent advancements in detection tasks. Additionally, the integration of the SSIM module facilitates data association, thereby enhancing the stability of target tracking while simultaneously reducing computational complexity and increasing the speed of target association.

## 2. Related Works

Traditional detection algorithms generally utilize a series of filters or contrast-based techniques for target identification. Du et al. [5] developed a method that integrates frame differencing with correlation filters to effectively track individual targets. Xuan et al. [6] improved the robustness of their tracking system against occlusion by combining correlation filters with Kalman filters and linear motion models. While these methodologies demonstrate promising results, they are primarily limited to single-target tracking, require initialization and predominantly focus on larger objects such as aircraft, trains, and automobiles. Wu et al. [7] introduced an innovative denoising technique that employs wavelet packet transform and kurtosis to detect dim and moving point targets within image sequences; however, the effectiveness of this approach diminishes when targets exhibit slow movement. In recent years, numerous contrast-based algorithms inspired by the human visual system (HVS) have emerged. For example, Kim et al. [8] developed an algorithm that enhances target visibility while suppressing background noise by optimizing the Target-to-Mean Clutter Ratio (TMSCR) within the Laplacian scale space. Nevertheless, this method tends to excessively amplify bright noise points, thereby increasing the false alarm rate. Han et al. [9] proposed an improved local contrast measurement algorithm for infrared images, referred to as the improved local contrast measure (ILCM), which reduced the high false alarm rate but was overly reliant on the sliding window parameter, resulting in decreased robustness. Subsequently, Nie et al. [10] introduced the Local Homogeneity Measure (LHM) for detecting dim and small infrared targets by evaluating local homogeneity and non-homogeneity across various scale templates. Although contrast-based detection algorithms can enhance detection accuracy compared to spatial filtering methods, their performance significantly deteriorates in complex backgrounds.

In recent years, advancements in deep learning within the field of visible light detection have facilitated the development of numerous high-performance algorithms for object detection. Compared to traditional detection methods, deep learning-based algorithms demonstrate enhanced adaptability and accuracy, notably, two-stage algorithms, including R-CNN (regions with convolutional neural network) [26], Fast R-CNN [27], and Faster R-CNN [28]. Additionally, single-stage algorithms, including the SSD (Single Shot MultiBox Detector) [29] and YOLO (You Only Look Once) [30,31], have gained significant attention. Two-stage algorithms delineate the processes of feature extraction and detection into distinct phases: initially identifying candidate target regions, followed by refining these regions to produce detection results. For instance, Du et al. [32] proposed a two-stage infrared target detection algorithm that first employed a convolutional neural network (CNN) for feature extraction, followed by classification using a Support Vector Machine (SVM), thereby achieving effective infrared target detection. Similarly, Sommer et al. [33] utilized the Region Proposal Network (RPN) from Faster R-CNN to generate candidate regions, which were subsequently classified by a CNN to determine their validity as targets. In contrast, one-stage algorithms directly infer class probabilities and bounding box coordinates to yield detection results, providing greater speed than two-stage algorithms by eliminating the need for extensive candidate box generation, thus rendering them more efficient for airborne platform applications. To address the limitations in shape and texture features of targets, Ding et al. [34] proposed an infrared small target detection algorithm based on the SSD Detector (SSD-ST). In subsequent studies [35,36], a multi-head attention mechanism was integrated into the YOLOv5s model to enhance small object detection, utilizing the CIoU loss function. When the detection of objects proves challenging, specific regional context can be leveraged to refocus attention on the object region [37]. For infrared targets characterized by limited pixel representation, an additional small-object detection layer was incorporated into the YOLOv5 model to achieve improved precision in small-object detection [38]. Zeng et al. [39] introduced an enhanced UAV image target detection algorithm based on YOLOv7, which effectively captured multi-scale feature information and improved model accuracy through the integration of the DpSPPF module. While these methodologies enhance detection performance by increasing focus on small objects or incorporating structures better suited for small-object detection, challenges such as missed detections, false alarms, and oversized models continue to persist.

Common baselines for online multi-object tracking include DeepSORT [11] and Deep OCSORT [12], which employ CNNs for detection, Kalman filters [16] for tracking, and re-identification (REID) modules to extract appearance features for multi-object matching. These methods have achieved significant results on multi-object tracking (MOT) datasets [40]. Wei et al. [17] utilized motion information, multiple Kalman filters, and various post-processing techniques to detect and track small objects in satellite videos. Despite the potential demonstrated by these methods, their performance often deteriorates when the Hungarian algorithm is applied as a post-processing step for data association, particularly in scenarios involving cluttered or low-texture infrared targets [41]. In our proposed approach, we advocate for the utilization of the GM-PHD filter for direct multi-object tracking, rather than relying on multiple separate Kalman filters. Unlike traditional multi-object tracking algorithms, the GM-PHD filter does not explicitly associate detected targets with tracked targets; instead, it estimates the number and states of targets through the probability hypothesis density function. This characteristic renders it particularly effective for tracking infrared targets that are characterized by weak-texture patterns.

## 3. Proposed Method

In this section, we will provide a detailed explanation of the proposed methodology for the detection and tracking of infrared weak-texture targets. The methodology consists of two primary components: a lightweight WTI-YOLO object detector integrated with a DSA module, and a GM-PHD multi-object tracker that employs SSIM for data association. We will begin with an overview of the overall framework of our approach, followed by a thorough analysis of each component.

### 3.1. Overview of Our Method

**Figure 2:**
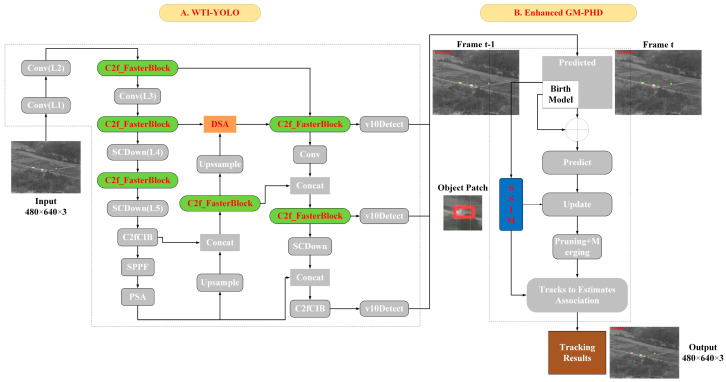
The comprehensive framework of the proposed algorithm. Enhancements were implemented utilizing the WTI-YOLO and GM-PHD algorithms to facilitate the detection and tracking of targets characterized by weak textures. The components for improving the detection model and tracking algorithm are highlighted in color, while other components are displayed in gray.

The proposed methodology for the detection and tracking of infrared weak-texture targets is organized into two distinct phases: single-frame target detection and tracking based on the GM-PHD filter. In the single-frame detection phase, we present an enhanced version of the YOLOv10n detection algorithm, designated as the WTI-YOLO. This model is specifically tailored to enhance the detection capabilities for infrared weak-texture targets. During the GM-PHD tracking phase, we consider the unique characteristics of infrared weak-texture targets and the imperative for real-time tracking by incorporating a SSIM module. This module captures the structural information of a 9 × 9 times the size around the target, thereby facilitating target association. This approach improves the accuracy of associating multiple targets in environments characterized by weak-texture features and complex backgrounds, while concurrently minimizing computational complexity to enhance tracking speed. The overall framework of the proposed methodology is depicted in Figure 2. Initially, the WTI-YOLO detector is executed on the current frame to identify all potential targets, resulting in detection outcomes. Subsequently, the GM-PHD filter is employed to eliminate results that deviate from the historical motion trajectories of the targets, thereby refining the detection results and determining the final positions of the targets within the current frame. Finally, the SSIM module is utilized to assist in distinguishing various targets between the estimated trajectories and detection boxes in frames k − 1 and k, ensuring accurate labeling of each target across the frames.

### 3.2. Target Detection Improvements

#### 3.2.1. DSA Module

In the realm of identifying infrared weak-texture targets within complex environments, object detection algorithms often face significant challenges related to instability during the processes of target extraction and recognition. This instability is primarily attributed to the weak shape and texture characteristics of targets in infrared imagery, which are further exacerbated by susceptibility to background noise. To mitigate these challenges and improve both detection accuracy and robustness, the present study introduces an innovative DSA module, which is integrated into the neck network of the WTI-YOLO object detection model, as depicted in Figure 2A. Specifically, the P3 feature layer in the backbone network is input into the Receptive-Field Attention (RFA) [42] submodule of the DSA module. This design allows for the adaptive capture of rich local target features within the shallow network, thereby enhancing the network’s ability to accommodate target deformations. Simultaneously, the PSA layer features, which contain deep semantic information, are transmitted through the neck network to the Efficient Multiscale Channel Attention (EMCA) [43] submodule of the DSA module. By adaptively adjusting channel weights, this approach effectively improves the network’s capacity to model long-range dependencies and extract global high-level semantic features. This design strategically integrates fine-grained information and edge features from shallow feature maps with high-level semantic information that represents the overall structure of the target from deep feature maps. As a result, the network can more effectively focus on key target areas, suppress redundant interference and background noise, and significantly enhance detection performance for weakly textured targets.

**Figure 3:**
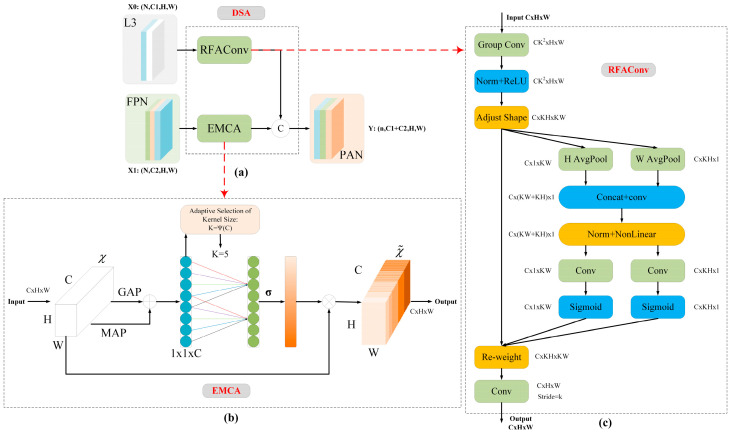
(**a**) The architecture of the DSA module. (**b**) EMCA module. (**c**) RFAConv module.

As depicted in Figure 3b, the EMCA module assigns weights to features across multiple channels, thereby enhancing the network’s ability to capture long-range dependencies. In contrast to traditional convolutional neural networks, this module demonstrates a reduced parameter count, which significantly improves computational efficiency while maintaining accuracy. Specifically, the input features are processed independently through AdaptiveAvgPool2d and AdaptiveMaxPool2d, followed by an additive operation that enriches the global perspective and accommodates features of varying scales. Subsequently, a one-dimensional convolutional layer is utilized to analyze the channels, resulting in a representation of inter-channel relationships. A Sigmoid activation function is then applied to normalize the results into attention weights, which are subsequently multiplied channel-wise with the original feature map to emphasize significant features while attenuating irrelevant information.

**Figure 4:**
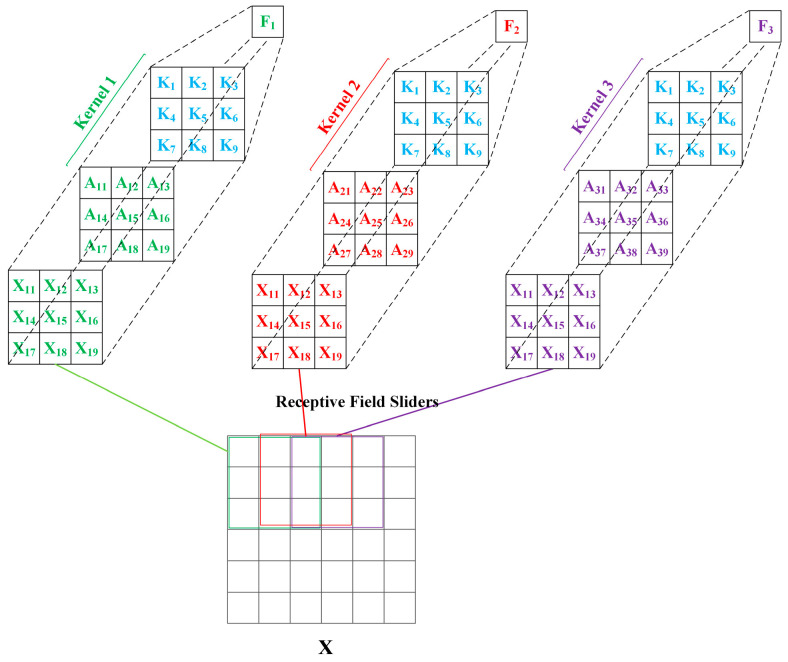
RFAConv module convolution operation process.

Figure 3c illustrates the RFA module, which incorporates the receptive field attention mechanism. This module generates distinct weights for each receptive field in the spatial dimension of the P3 feature layer X, enabling the network to assign varying weights to each receptive field, resulting in the production of a new feature map F. As evidenced in Figure 4, during the convolution process, the parameters of the convolution kernel are obtained by multiplying the attention weights with the kernel parameters K, resulting in a customized convolution kernel for each receptive field position. For example, in the figure, Kernel 1, Kernel 2, and Kernel 3 are produced by multiplying the general convolution kernel parameters K with their corresponding attention weights A_1_, A_2_ and A_3_. This approach allows the network to assign varying degrees of importance to features at different spatial locations during the feature extraction process, thereby enhancing its ability to capture critical features.

Notably, the spatial receptive field attention mechanism and the multiscale channel attention mechanism within the DSA module do not operate independently; rather, they achieve efficient integration. This distinctive structural design empowers the model to effectively enhance critical details associated with targets while simultaneously suppressing irrelevant features, thereby further improving the detection accuracy of infrared targets.

#### 3.2.2. C2f_Fasterblock Module

**Figure 5:**
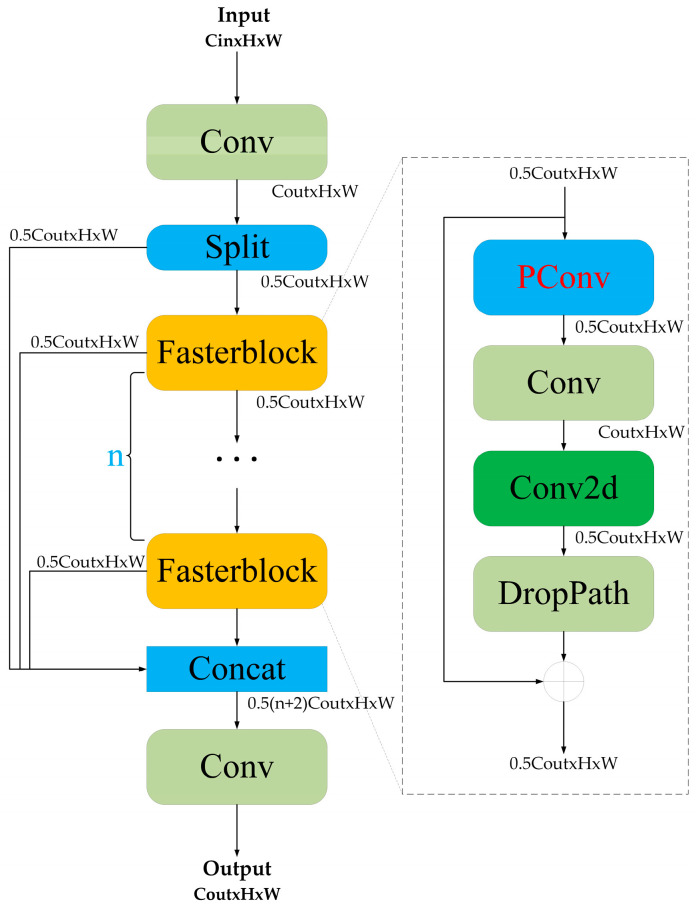
The C2f_Fasterblock mdule.

Although the implementation of the previous DSA module has enhanced the detection accuracy of the network, this improvement comes with an increase in the number of parameters, which subsequently led to a reduction in detection speed. Considering the limitations of computational resources available in airborne devices, the C2f_FasterBlock module [44] was introduced. This module improves upon the original C2f residual architecture of YOLOv10, resulting in a significant reduction in both the total number of parameters and the computational requirements of the network by replacing the original design. The specific configuration of the C2f_FasterBlock module is depicted in Figure 5.

**Figure 6:**
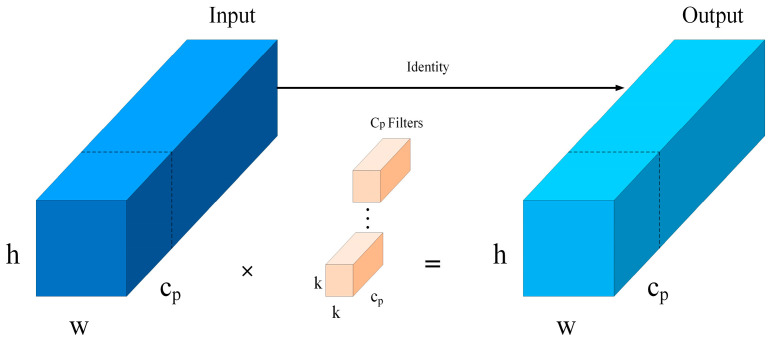
The Partial Convolution schematic diagram.

The FasterBlock module in the main branch maintains the original dual-branch architecture while integrating DropPath and Partial Convolution (PConv) [45] to reduce computational load and improve resilience against target occlusion and environmental interference. Specifically, DropPath introduces path-level randomness by randomly omitting certain paths in the network, thereby enhancing the model’s structural diversity and generalization capability, which effectively mitigates overfitting. In contrast, PConv limits the convolution operation to a specific subset of channels within the feature map, whereas traditional convolution operations typically apply to all channels of the input feature map. This approach effectively reduces unnecessary memory access load and computation by omitting portions of the input deemed redundant. As illustrated in Figure 6, the variables w and h represent the width and height of the output and input feature maps, respectively; Cp denotes the number of channels involved in the convolution; and k indicates the kernel size. Cp and k represent the number of convolution channels and the kernel size, respectively. The PConv method selectively extracts spatial features from a specific subset of the input feature map channels, while preserving the remaining channels, which are subsequently passed to the following layer without modification. The selection of either the initial or final continuous channels for memory storage of the complete feature map can enhance the efficiency of memory access. When the number of channels in both the input and output feature maps is identical, the computational complexity of the PConv module can be calculated using Equation (1).

(1) $$h\times w\times k^{2}\times c_{p}^{2}.$$

Moreover, the PConv demonstrates a relatively low demand for memory access, as indicated in Equation (2).

(2) $$h\times w\times 2c_{p}+k^{2}\times c_{p}^{2}\approx h\times w\times 2c_{p}.$$

The C2f_FasterBlock module introduces a multi-branch architecture across stages, allowing for the partial processing of input features through various branches. One branch enhances the network’s ability to express nonlinearity by stacking multiple FasterBlock modules, while the other branch preserves some of the original features, ultimately merging the two. This design strikes a balance between depth and width, improving the effectiveness of feature extraction while mitigating issues related to information loss and network degradation.

### 3.3. GM-PHD Tracking Improvements

#### 3.3.1. The SSIM Module

The GM-PHD filter demonstrates the capability to differentiate between genuine and spurious targets; however, it is limited in its ability to distinguish between two distinct targets. Therefore, supplementary methodologies are required to identify separate targets across successive frames.

**Figure 7:**
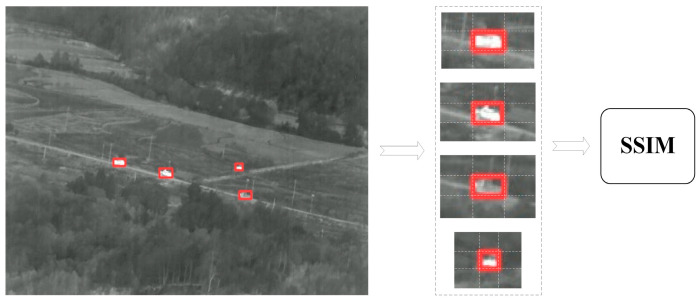
Image patches of 9 × 9 times the size around the target.

In this study, we propose a structural similarity (SSIM) module that evaluates visual affinities between image patches measuring 9 × 9 pixels surrounding the target, as depicted in Figure 7, rather than employing a convolutional neural network (CNN)-based re-identification (REID) module for the extraction of target feature information. The SSIM module is primarily utilized to assess the similarity between two images of identical dimensions. Although infrared targets often exhibit minimal shape and texture characteristics, the inclusion of their surrounding environment provides particularly informative texture data. Compared to the CNN REID module, the application of the SSIM module improves the accuracy of multi-object association while significantly reducing computational complexity and enhancing the speed of target association.

The SSIM algorithm evaluates the similarity between two image patches, referred to as α and β, by examining their luminance, contrast, and structural components. It subsequently combines these three elements using a weighted approach, which is expressed as a multiplicative formula given by

(3) $$SSIM(\alpha ,\beta )=\frac{\left(2\sigma_{\alpha \beta }+c_{1})(2\mu_{\alpha }\mu_{\beta }+c_{2}\right)}{\left(\sigma_{\alpha }^{2}+\sigma_{\beta }^{2}+c_{1})(\mu_{\alpha }^{2}+\mu_{\beta }^{2}+c_{2}\right)}.$$

where $\sigma_{\alpha },\sigma_{\beta },\mu_{\alpha },\mu_{\beta }$ and $\sigma_{\alpha \beta }$ represent the variance, mean, and covariance of patches α and β, respectively. $c_{1}={\left(k_{1}I\right)}^{2}$ and $c_{2}={\left(k_{2}I\right)}^{2}$ are to avoid division by zero, where I represents the maximum pixel value. By default, $k_{1}$ = 0.01 and $k_{2}$ = 0.03.

Let $b_{m,k-1}^{t}$ and $b_{n,k}^{d}$ denote the estimated position of the $m^{th}$ tracklet and the $n^{th}$ detection box position at frame k, respectively. The spatiotemporal similarity is obtained using the traditional Euclidean distance $D_{k}(b_{m,k-1}^{t},b_{n,k}^{d})$, defined as follows:

(4) $$D_{k}(b_{m,k-1}^{t},b_{n,k}^{d})=\sqrt{{\left(b_{m,i,k-1}^{t}-b_{n,j,k}^{d}\right)}^{2}+{\left(b_{m,i,k-1}^{t}-b_{n,j,k}^{d}\right)}^{2}}.$$

where $\left(b_{m,i,k-1}^{t},b_{m,j,k-1}^{t}\right)$ and $\left(b_{n,i,k}^{d},b_{n,j,k}^{d}\right)$ represent the center positions of their respective bounding boxes in frames k and k−1.

#### 3.3.2. Tracking Strategy

The method proposed in this study is based on Random Finite Set (RFS) [46] modeling of the states and observations of multiple targets. At frame k, the set of target states is represented as $X_{k}=\{x_{k}^{1},x_{k}^{2},\ldots ,x_{k}^{M_{k}}\}$, where $M_{k}$ denotes the actual number of targets present in the current frame. Correspondingly, the set of observations is represented as $Z_{k}=\{z_{k}^{1},z_{k}^{2},\ldots ,z_{k}^{N_{k}}\}$, where $N_{k}$ indicates the number of observations generated by the detector in that frame.

**Figure 8:**
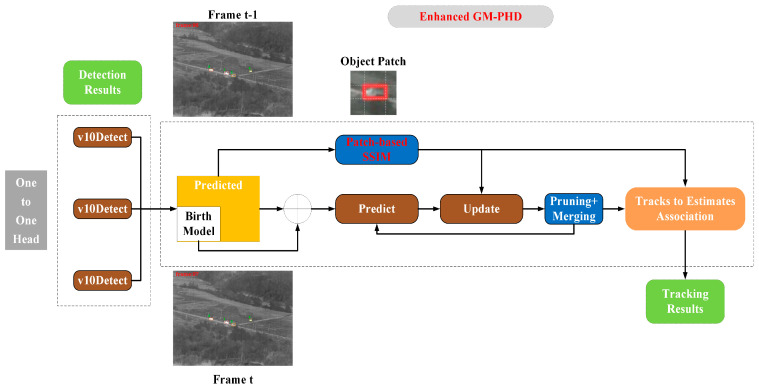
GM-PHD tracking framework enhanced by SSIM module.

As illustrated in Figure 8, the complete multi-target tracking process is as follows: First, the WTI-YOLO detector is utilized to obtain the target detection results for the current frame, which serve as the observational input for subsequent tracking tasks. The GM-PHD filter is employed to approximate the modeling of target states, represented in a weighted Gaussian mixture form, expressed as follows:

(5) $$D_{k}(x)=\overset{J_{k}}{\underset{j=1}{\Sigma }}w_{k}^{j}Ν(x;m_{k}^{j},P_{k}^{j}).$$

where $w_{k}^{j},m_{k}^{j}$ and $p_{k}^{j}$ are the weight, mean, and covariance for the $j^{th}$ component and $J_{k}$ is the number of components.

Next, by combining the tracking results from the previous frame, a prior state for the current frame is generated through a prediction step, defined as follows:

(6) $$D_{k|k-1}(x)=\lambda (x)+\alpha_{s}\overset{J_{k-1}}{\underset{j=1}{\Sigma }}w_{k-1}N(x;Fm_{k-1}^{j},Q+FP_{k-1}^{j}F^{T}).$$

where F and Q are the transition and motion covariance matrices in the same format as in [47], λ(x) is the birth RFS intensity, and $\alpha_{s}$ is a hyper-parameter to denotes the survival probability.

On this basis, the observation information from the current frame is fused to perform an update. The “Pruning + Merging” module optimizes Gaussian components by trimming and merging them according to their weights, thereby reducing computational complexity. The update process can be expressed as follows:

(7) $$D_{k}(x)=(1-p_{D,k})D_{k|k-1}(x)+\underset{z\in Z_{k}}{\Sigma }\overset{J_{k|k-1}}{\underset{j=1}{\Sigma }}w_{k}^{j}(z)N(x;m_{k|k}^{j}(z),P_{k|k}^{j}).$$

where $D_{k|k-1}(x)$ denotes the predicted GM components and β is a hyper-parameter to denote the detection probability. The terms $m_{k|k}^{j}(z),P_{k|k}^{j}$ are the target–measurement association mean and covariance, which are calculated using the Kalman equations, and $w_{k}^{j}(z)$ denotes the updated weight for the data association and is defined as:

(8) $$w_{k}^{j}(z)=\frac{s_{k}p_{D,k}w_{k|k-1}^{j}q_{k}^{j}(z)}{c_{sk}(z)+p_{D,k}\Sigma_{i=1}^{J_{k|k-1}}w_{k|k-1}^{i}q_{k}^{i}(z)}.$$

where $c_{sk}(z)$ denotes the clutter process intensity and $q_{k}^{j}(z)$ denotes the target–measurement association likelihood. $s_{k}=SSIM(\alpha ,\beta )$ represents visual similarity, which is calculated by the SSIM module.

Finally, the “Tracks to Estimates Association” step completes the data association between state estimation and tracking trajectories. The proposed method utilizes Munkres’s variant of the Hungarian algorithm [48], combined with the structural similarity index (SSIM) for appearance similarity and spatiotemporal similarity, to construct the following association cost function. This function determines the optimal matching relationship of targets across frames.

(9) $$C_{k}=\eta D_{k}+(1-\eta )S_{k}.$$

where $S_{k}\in [0,1]$, $D_{k}\in [0,1]$, with $S_{k}$ denoting visual similarity, $D_{k}$ being normalized, and the variable η employed to equilibrate the two costs in accordance with tracking performance and where $C_{k}\in R^{N\times M},D_{k}\in R^{N\times M}$, and $S_{k}\in R^{N\times M}$, with N and M representing the number of detections and estimates at frame k.

The spatiotemporal relationship offers critical insights into the association of tracklets corresponding to targets that are in close proximity; however, its relevance diminishes as the temporal distance between targets increases. In contrast, the visual similarity derived from the SSIM module supports long-range associations, demonstrating resilience to significant temporal and spatial separations. The integration of spatiotemporal and visual information assists in resolving ambiguities related to target identification, which may arise from either the movement of the targets or their visual characteristics, while also facilitating long-range target associations. The filtered detection boxes are subsequently appended to their respective tracklets, thereby extending them up to time k

## 4. Experiment

### 4.1. Dataset and Implementation

The present experiment was conducted on a Linux system, specifically Ubuntu 20.04, and the programming was based on Python 3.8. PyTorch 1.10 was used as the algorithm framework, supplemented by CUDA 11.3 and cuDNN 8.2 to enhance the efficiency of model training. The hardware configuration comprised an Intel Core i9-9900K CPU along with an NVIDIA RTX3090 graphics card. The training process utilized the AdamW optimizer, with the learning rate gradually reduced from an initial value of 0.01, a momentum value of 0.937, and a weight decay of 0.005. At the same time, the task decoupling loss function was utilized to model and optimize the three tasks of classification, regression, and object existence in object detection separately. A dynamic label assignment strategy was implemented to select positive and negative samples, enabling each sub-task to train on the most appropriate samples. Model training was performed using a batch size of 16 for a total of 300 epochs, with early stopping criteria determined after 50 iterations of training; this criterion dictated that training would cease if there was no improvement in model performance over 50 consecutive epochs. Other parameters remain consistent with the default settings of the original YOLOv10n model.

**Figure 9:**
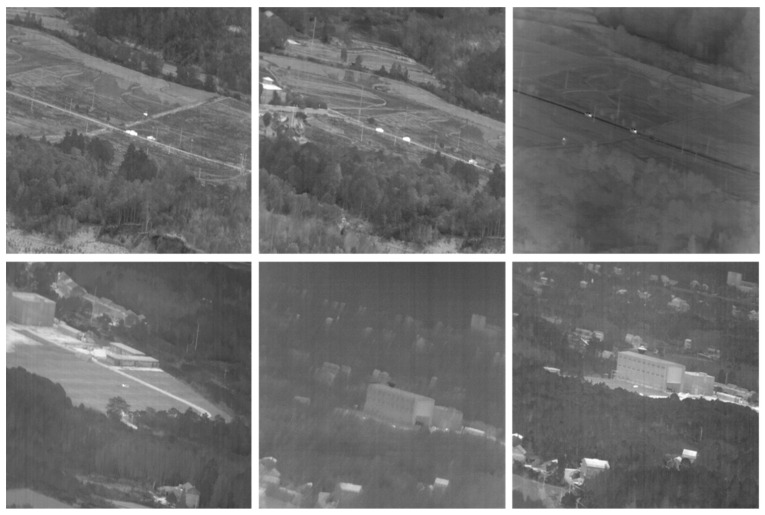
Typical images of different sequences in the dataset.

The dataset utilized in this experiment has been specifically curated for the purpose of infrared time-sensitive target detection and tracking, with a focus on ground–air applications. It encompasses a total of 87 video sequences, which include 89,174 targets, 393 target trajectories, and 21,750 frames of images. To ensure a randomized and comprehensive training process, the dataset has been partitioned into training, validation, and test sets in a ratio of 7:1:2. The training process utilized and enhanced classic YOLO series data augmentation techniques, including Mosaic; MixUp; color space perturbations; and fundamental transformations such as random cropping, scaling, and flipping. Additionally, a dynamic augmentation scheduling strategy was implemented to adaptively adjust the strength of augmentations throughout various stages of training. Figure 9 presents typical images of different sequences from the dataset.

### 4.2. Evaluation Metrics

The assessment of the algorithm presented in this article primarily focuses on two key aspects: object detection and multi-object tracking. The following evaluation metrics were employed to assess the algorithm’s performance across various dimensions:

● **Object detection evaluation:** Mean average precision (mAP) is defined as the average of the average precision (AP) across various categories, where AP is calculated as the area under the precision–recall (P−R) curve, a higher mAP signifies that the model not only maintains high precision but also exhibits robust recall performance.
  (10) $$mAP=\frac{1}{N}\overset{n}{\underset{i=1}{\Sigma }}AP_{i},$$
  where $AP_{i}$ is given by
  (11) $$AP=\int_{0}^{1}P(r)dr,$$
  (12) $$P=\frac{TP}{TP+FP},$$
  (13) $$R=\frac{TP}{TP+FN},$$
  where TP (True Positive) and FP (False Positive) denote the number of samples that the model accurately and inaccurately predicted as positive, respectively. Conversely, FN (False Negative) refers to the number of samples that the model incorrectly classified as negative.

Additionally, Floating Point Operations per Second (FLOPs) and Frames Per Second (FPS) are crucial indicators to determine whether the model can satisfy real-time operational requirements, as described in the following formula:

(14) $$FPS=\frac{N}{T}.$$

where the total number of images processed is denoted as N, and the overall processing time is represented by T.

● **Multi-object tracking evaluation:** MOTA is one of the CLEAR [49] metrics employed to assess the overall accuracy of multi-object tracking algorithms. This metric considers three primary types of errors: ID switches, FP, and FN. The calculation of MOTA is performed using the following formula:
  (15) $$MOTA=1-\frac{\Sigma_{f}(IDS_{f}+FN_{f}+FP_{f})}{\Sigma_{f}GT_{f}}.$$
  where $IDS_{f}$ denotes the number of identity switches per frame, while $GT_{f}$ signifies the total number of ground truth targets per frame.

Nevertheless, due to the limitations of the CLEAR metric in assessing identity switches across multiple targets, the IDF1 metric places greater emphasis on the accurate tracking of the complete trajectory of each target. Consequently, the incorporation of the IDF1 [50] metric provides a more intuitive representation of the quality of associations throughout the tracking process.

(16) $$IDF1=\frac{2IDP\cdot IDR}{IDP+IDR}.$$

where IDP represents the proportion of correctly identified target identities in the tracker output, while IDR measures the proportion of true identities that the tracker correctly identifies. IDF1 is the harmonic mean of IDP and IDR. Compared to MOTA, IDF1 provides a better measure of the consistency of ID matching.

### 4.3. Evaluation of Experimental Results

#### 4.3.1. Detection Experiment

##### Ablation Experiment

This section of the study evaluates the proposed WTI-YOLO through a series of ablation experiments designed to assess the effectiveness of various components within the model. The primary objective of these experiments is to ascertain the impact of each module on the performance of WTI-YOLO. To ensure the reliability of the results, all experiments are conducted under controlled conditions. In accordance with the guidelines established by the infrared tracking dataset, image sequences 1–76 are allocated to the training set, while sequences 77–87 are utilized as the test set. The results of the experiments conducted on the infrared tracking dataset are summarized in Table 1.

Table 1 demonstrates that the optimized WTI-YOLO model exhibits improved detection precision and recall, as well as a reduction in model size and FLOPS when compared to the original network architecture. This optimization leads to an increased detection frame rate on the GPU. The integration of the EMCA module resulted in a 0.75% improvement in precision, a 0.64% enhancement in recall, and a 0.73% increase in mAP@0.5. These findings suggest that the module effectively captures more complex patterns and high-level semantic information, thereby enabling the network to focus on critical features and enhancing the detection accuracy of targets with weak textures. Moreover, the incorporation of the RFAConv module achieved a detection recall of 96.6% and an mAP@0.5 of 98.2%. This indicates that the module can adaptively adjust the attention regions on shallow feature maps that are rich in detailed features, thereby allowing the model to more accurately identify target areas and improve detection robustness. Following these enhancements, the introduction of the DSA module further increased detection precision to 94.5%, recall to 96.8%, and mAP to 98.1%, resulting in a highly accurate detection network that meets the demands of real-world applications. Considering the computational constraints of airborne devices, the implementation of the C2f_FasterBlock module led to a 16.3% reduction in the number of parameters and an 11.0% decrease in computational complexity. The model achieves a detection frame rate of 113 FPS on the GPU, demonstrating its capability to meet real-time detection requirements for computationally limited airborne devices and significantly enhancing the efficiency of subsequent multi-object tracking tasks.

##### Robustness Testing in Different Environments

The video sequences in the infrared tracking dataset encompass two typical road surface scenarios: unstructured road scenarios featuring a relatively open road environment and structured road scenarios characterized by a complex background. In comparison, the structured road scenarios exhibit greater background interference, making them susceptible to issues such as target occlusion and multi-target intersections. Furthermore, data collection was performed under two distinct lighting conditions: during the day and in the evening. The evening environment presents a lower overall temperature and reduced contrast, while the daytime scenarios offer higher brightness and clarity. To thoroughly evaluate the robustness of the proposed algorithm across various environmental conditions, systematic tests were conducted to assess detection performance under the aforementioned scene and lighting combinations, with the experimental results presented in Table 2.

From Table 2, it is evident that the proposed algorithm performs optimally in unstructured road daytime scenarios, achieving a mAP of 98.5%. In structured road daytime and unstructured road evening (low light) conditions, the performance experiences a slight decline, with the mAP decreasing by approximately 0.4% compared to the peak value. This indicates that the model demonstrates good robustness across various environmental conditions. However, there remains potential for improvement in adaptability under low light and complex surface backgrounds. Further analysis reveals that in unstructured road evening scenarios, the detection accuracy decreases by 5.3%, indicating a more pronounced performance drop. These results suggest that future work could benefit from the introduction of more targeted data augmentation strategies or the adoption of domain adaptation methods to further enhance the model’s generalization capabilities in non-ideal environments.

##### Comparison of Advanced Detection Models

In this section, we present a series of comparative experiments aimed at evaluating the efficacy of WTI-YOLO in the context of infrared ground target detection tasks, utilizing an infrared tracking dataset. Currently, there are two main categories of traditional single-stage infrared target detection models: the SSD series and the YOLO series of advanced algorithms. The YOLO models, which benefit from advanced network architectures and efficient feature extraction techniques, are particularly advantageous for applications that require both high detection speed and accuracy. In contrast, SSD models are characterized by a more straightforward architecture, conducting object detection directly on feature maps of varying scales. While SSD demonstrates superior performance in detecting medium and large targets, its accuracy in identifying small targets and managing complex backgrounds is generally inferior to that of YOLO models, especially when analyzing low-resolution images. To facilitate a comprehensive comparison, we selected several state-of-the-art detection algorithms based on SSD and YOLO that have emerged in recent years for evaluation. The findings are presented in Table 3. To ensure a fair comparison, this experiment employs the same training and testing set partitioning strategy as outlined in [44], wherein the 87 image sequences are divided such that odd-numbered sequences are designated as the training set, while even-numbered sequences are allocated to the test set. In Table 3, methods 1–4 are categorized under the YOLO series, whereas methods 5–7 are derived from the SSD framework.

The findings indicate that the proposed algorithm exhibits exceptional detection capabilities when assessed using the infrared tracking dataset. The model that attained the highest level of detection accuracy is SSD-ST, which achieves a precision of 97.4%. Closely following is our enhanced model, WTI-YOLO, which attains a precision of 97.3%, only 0.1% lower than the highest accuracy. Furthermore, WTI-YOLO shows a 22% improvement in recall rate compared to SSD-ST. In comparison to IRSDet, it also achieves a 0.8% enhancement, resulting in the best overall performance. These findings suggest that our model is effective in detecting infrared weak-texture targets.

**Figure 10:**
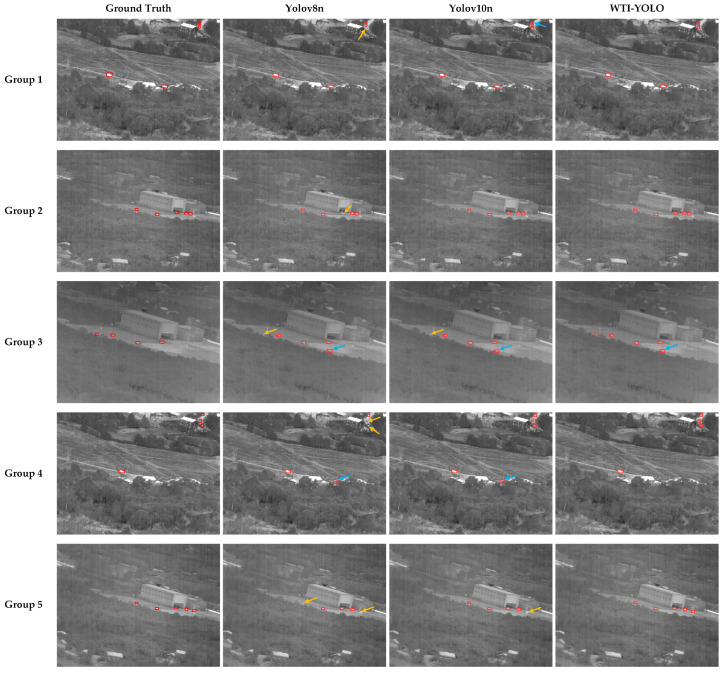
Visualization comparison of detection results from different algorithms. The red box indicates the detected infrared target. The yellow arrows represent missed detections, while the blue arrows represent false detections.

In addition, to intuitively compare the improvements in detection performance of the WTI-YOLO model, which is based on YOLO, for weak-texture target detection, five images from different scenes in the infrared tracking dataset were selected. The detection performance of the YOLOv8n, YOLOv10n, and WTI-YOLO models was evaluated. The detection results are presented in Figure 10. Each set of images includes the ground truth, followed by the detection results from YOLOv8n, YOLOv10n, and our WTI-YOLO model. Missed detections are indicated with yellow arrows, while false detections are marked with blue arrows.

From the results presented in the Figure 10, it is evident that the proposed model exhibits lower missed detection rates and false detection rates compared to other algorithms in complex environments. This indicates that the proposed improvement strategy demonstrates good adaptability and robustness in infrared scenarios. In the fourth group of samples, YOLOv8n displays a significant missed detection phenomenon in areas with occlusion at the edges of the scene. Additionally, both YOLOv8n and YOLOv10n are affected when environmental heat sources are partially obscured by trees, resulting in false detections. In contrast, WTI-YOLO consistently detects all actual targets, showcasing strong adaptability to occlusion. Furthermore, in the fifth group, during the unstructured road evening (low light) scene, changes in environmental temperature led to a decrease in image contrast, causing other detection models to exhibit varying degrees of missed detections. In contrast, WTI-YOLO continues to accurately detect all targets, further validating its robustness and detection performance in low-contrast infrared images. Nevertheless, in the third group of images under the same conditions, all algorithms displayed false detections. In summary, compared to other detection algorithms, WTI-YOLO demonstrates superior target detection capabilities in complex infrared environments, significantly reducing the occurrence of missed detections and false detections, thereby confirming its effectiveness in infrared small target detection tasks.

#### 4.3.2. Tracking Experiment

##### Comparison to MOT Methods

This section provides an overview of a range of advanced multi-object tracking (MOT) algorithms employed for target tracking within the infrared tracking dataset. To ensure consistency, all algorithms utilize the detection model developed in this study. The final performance metrics for tracking are obtained from a consistent test set, with the findings detailed in Table 4 below.

A comprehensive analysis of the various metrics presented in Table 4 indicates that the proposed enhanced algorithm effectively addresses several challenges associated with tracking weak-texture infrared targets. It successfully reduces ground clutter interference in complex scenarios and platform motion interference. As a result, the algorithm demonstrates significant performance improvements across all evaluated metrics. Notably, a score of 90.7% was achieved on the MOTA metric, with the number of IDs reduced to single digits compared to the suboptimal StrongSORT algorithm. The FN metric was significantly reduced by approximately four times. This demonstrates that optimizing the detector greatly decreased the number of missed detections in object detection, which also helped to minimize trajectory interruptions caused by these missed detections, promoting a more continuous and comprehensive tracking path. The key metric IDF1 achieved a score of 94.6%, representing an improvement of 11.4% over the suboptimal Deep OCSORT algorithm, while the IDP and IDR metrics outperformed those of other algorithms. This finding indicates that the optimized detection algorithm for weakly textured targets, combined with data association based on SSIM during the tracking phase, significantly enhances tracking stability and further increases the practical applicability of the tracking results generated by the model. Additionally, the lightweight optimization of the detector network and the integration of the SSIM module within the tracking algorithm result in a considerable reduction in overall computational load, achieving a tracking speed of 102 FPS. This indicates that the proposed algorithm not only maintains high tracking performance but also achieves a more efficient and streamlined structural design.

##### Testing Tracking Performance in Different Environments

To thoroughly assess the tracking performance of the proposed algorithm across diverse environmental conditions, systematic tracking experiments were conducted using various combinations of scenes and lighting conditions, as detailed in the section Robustness Testing in Different Environments. The pertinent experimental results are summarized in Table 5.

The experimental results indicate that the proposed detection and tracking algorithm performs optimally in well-lit, unstructured road scenarios during the day, achieving MOTA and IDF1 scores of 90.1% and 94.8%, respectively. However, under non-ideal conditions, such as complex backgrounds in structured road scenarios and low-light unstructured road scenarios, the algorithm’s performance exhibits a noticeable decline, evidenced by an increase in identity switch frequency and the number of false detections. This suggests that there is still room for improvement in robustness within complex environments. Overall, this method demonstrates strong generalization ability and adaptability in most typical environments, highlighting its potential and feasibility for deployment in real-world variable conditions.

##### Qualitative Analyses

**Figure 11:**
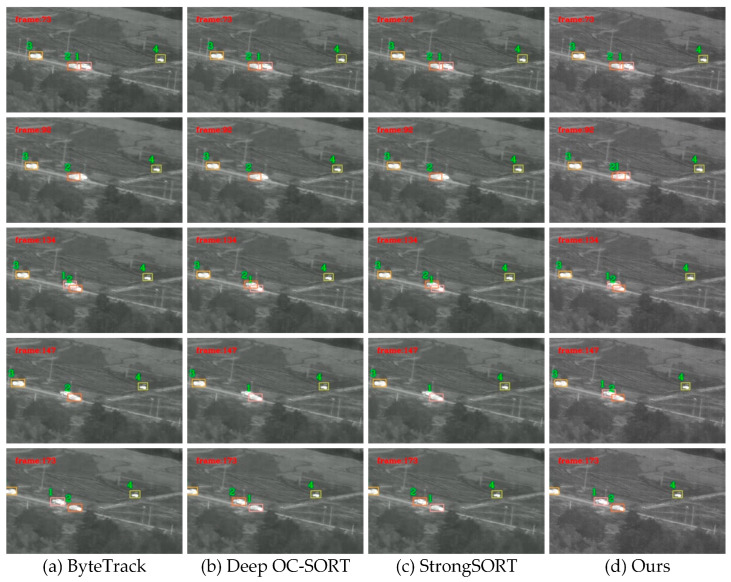
Qualitative experimental results. The target ID is represented in numerical order.

In addition to quantitative assessments, we have visualized the experimental outcomes across several sequences. Figure 11 presents the partial tracking results for ByteTrack, Deep OC-SORT, StrongSORT, and the proposed tracker within the infrared trcking dataset. At frame 92, target ID1 is partially obscured by target ID2. The first three methodologies fail to maintain tracking of target ID1, whereas the proposed algorithm successfully continues tracking. Upon the reappearance of the target at frame 134, the other methods exhibit ID switching between target ID1 and ID2, in contrast to the proposed algorithm, which does not demonstrate such behavior. Consequently, when compared to alternative methods, the proposed approach exhibits superior performance in terms of continuity, thereby reducing missed detections and false detections within complex infrared environments.

## 5. Conclusions

This research presents a real-time detection and tracking algorithm specifically designed for the identification and monitoring of infrared weak-texture targets within complex environments. Initially, an enhanced object detection model, referred to as WTI-YOLO, was employed to identify infrared weak-texture targets in high-resolution imagery. To effectively address the unique characteristics of infrared weak-texture targets in conjunction with background information, a deep–shallow attention module was developed. Subsequently, the GM-PHD tracking algorithm, which is proficient in managing scenarios involving a variable number of targets, was implemented for the tracking of infrared weak-texture targets. This approach provides an efficient multi-object tracking solution that is specifically tailored for infrared weak-texture targets, achieving a commendable balance between real-time performance and accuracy. The effectiveness of the proposed method was validated against publicly available infrared tracking datasets.

## Figures and Tables

**Table 1:** Results of ablation experiments on WTI-YOLO.

Yolov10n	DSA		C2f_Fasterblock	P (%)	R (%)	mAP0.5 (%)	Params (M)	FLOPs (G)
	EMCA	RFAConv						
√				93.8	93.7	95.9	2.69	8.2
√	√			94.5	94.3	96.6	2.74	8.2
√		√		94.5	96.6	98.2	2.74	8.8
√	√	√		94.5	96.8	98.1	2.74	8.8
√	√	√	√	**96.0**	**97.3**	**98.5**	**2.25**	**7.3**

Bold number: optimal result.

**Table 2:** Robustness testing of detection performance under different scenarios and lighting combinations.

Road Scenarios	Lighting Conditions	Video Sequences	TP	FN	FP	P (%)	R (%)	mAP0.5 (%)
structured road	daytime	78–80	2275	55	65	97.2	**97.6**	98.1
unstructured road	daytime	81–83	3627	121	83	**97.8**	96.8	**98.5**
	evening	84–86	3590	95	291	92.5	97.4	98.1

Bold number: optimal result.

**Table 3:** Comparison of various infrared target detection algorithms.

No.	Models	TP	FN	FP	P (%)	R (%)	Params (M)	FLOPs (G)
1	SSD-ST [34]	33,512	11,531	895	**97.4**	74.4	2.8	24.8
2	FD-SSD [51]	29,683	15,360	1013	96.7	65.9	5.8	30.1
3	DF-SSD [52]	29,404	15,639	1007	96.7	65.3	10.0	31.6
4	IRSDet [53]	40,539	4504	1383	96.7	90.0	7.7	19.8
5	Yolov8n [54]	39,654	5389	2201	94.7	88.0	3.0	8.1
6	Yolov10n [22]	39,335	5708	2029	95.1	87.3	2.7	8.2
7	WTI-YOLO (ours)	470,916	4127	1141	97.3	**90.8**	**2.2**	**7.3**

Bold number: optimal result.

**Table 4:** Test results of various infrared multi-object tracking algorithms on the server.

Tracker	IDs↓	MOTA↑	Frag↓	FP↓	FN↓	IDR↑	IDP↑	IDF1↑	FPS↑
DeepSort [11]	113	80.2	300	491	1513	71.4	79.0	75.0	90
OC-SORT [13]	32	79.1	163	345	1851	77.3	87.1	81.9	95
Deep OC-SORT [12]	17	80.5	175	380	1693	79.7	90.8	84.9	82
ByteTrack [14]	35	81.0	193	413	1584	76.1	85.4	80.5	80
StrongSORT [15]	74	82.0	190	399	1457	78.5	87.6	82.6	79
Ours	**2**	**90.7**	173	670	328	96.1	93.2	**94.6**	**102**

Bold number: optimal result. Use arrows to indicate the optimal direction of change for each indicator.

**Table 5:** Tracking performance under different scenarios and lighting combinations.

Road Scenarios	Lighting Conditions	Video Sequences	IDs↓	MOTA↑	Frag↓	FP↓	FN↓	IDR↑	IDP↑	IDF1↑
structured road	daytime	78–80	0	87.7	59	174	112	95.9	92.3	94.0
unstructured road	daytime	81–83	1	**90.1**	61	170	130	95.9	93.7	**94.8**
	evening	84–86	1	88.3	31	295	56	96.2	90.0	92.7

Bold number: optimal result. Use arrows to indicate the optimal direction of change for each indicator.

## Data Availability

Datasets Link: http://www.doi.org/10.11922/sciencedb.j00001.00331 (accessed on 1 November 2024).
